# Neurobrucellosis: a retrospective cohort of 106 patients

**DOI:** 10.1186/s41182-025-00680-1

**Published:** 2025-01-15

**Authors:** Fatemeh Arazi, Mahboubeh Haddad, Fereshte Sheybani, Mohammad Taghi Farzadfard, Majid Khadem Rezaeian

**Affiliations:** 1 Department of Infectious Diseases and Tropical Medicine, Faculty of Medicine, Mashhad University of Medical Sciences, Mashhad, Iran; 2 Department of Neurology, Faculty of Medicine, Mashhad University of Medical Sciences, Mashhad, Iran; 3 Department of Community Medicine, Faculty of Medicine, Mashhad University of Medical Sciences, Mashhad, Iran

**Keywords:** Neurobrucellosis, Brucella species, Nervous system infection, Mortality, Survival probability

## Abstract

**Background:**

Neurobrucellosis, a serious central nervous system infection caused by Brucella species, presents significant challenges due to its diverse clinical manifestations and the risk of long-term complications and poor outcomes. Identifying predictors of adverse outcomes is critical for improving patient management and overall prognosis.

**Objectives:**

This study aimed to evaluate the long-term morbidity and mortality associated with neurobrucellosis and to identify key predictors of adverse outcomes.

**Methods:**

We performed a retrospective cohort study of 106 neurobrucellosis patients treated at two major referral centers in Mashhad, Iran, from March 21, 2011, to March 20, 2022. We analyzed clinical, neuroimaging, and laboratory data, and estimated survival probabilities using Kaplan–Meier analysis. Long-term morbidity was evaluated using the Glasgow Outcome Scale.

**Results:**

The median age of the cohort was 30 years (IQR: 21.8–46.3). The median length of hospital stay was 11 days (IQR: 7–19.8), with an in-hospital mortality rate of 4.7% (n = 5). Survival probabilities were 92.2% (SE = 0.027) at 1 month and 90.1% (SE = 0.030) at 6 months. The median follow-up duration was 52 months (IQR: 35–77). At follow-up, 23.5% (n = 20) of patients had an unfavorable outcome based on the Glasgow Outcome Scale. Predictors of mortality included older age, altered level of consciousness, seizures, elevated body temperature on admission, and white matter changes on neuroimaging.

**Conclusion:**

Neurobrucellosis is associated with significant long-term morbidity and mortality. Key predictors of mortality include older age, altered level of consciousness, seizures, elevated body temperature on admission, and white matter changes. Identifying these predictors can help in targeting therapeutic strategies and improving patient outcomes through early intervention and close monitoring.

**Supplementary Information:**

The online version contains supplementary material available at 10.1186/s41182-025-00680-1.

## Introduction

Human brucellosis remains a significant global health challenge despite progress in some regions. While its incidence has declined in Western Europe and the United States, and eradicated in some countries, it remains one of the most widespread zoonotic diseases. It is endemic in areas like the Middle East, Africa, and parts of South America [1]. In Iran, the disease continues to rise, with reported cases increasing from 88,450 in 2009 to 198,030 in 2015, while the mortality rate has risen from 244 to 578 over the same period [2]. Transmission primarily occurs through consuming contaminated food or unpasteurized dairy products, contact with infected animals via wounds, or inhaling aerosols [3].

Human brucellosis is a multisystem disease that can affect various organs, leading to a wide range of clinical manifestations. When the nervous system is involved, it is referred to as neurobrucellosis [4]. This condition is rare, affecting 3–10% of patients with brucellosis and constituting approximately 0.5% of community-acquired central nervous system (CNS) infections worldwide [1]. Neurological complications from Brucella infection are particularly infrequently reported in children, with only 0.8% of those with systemic brucellosis experiencing these issues [4]. Despite its relative rarity, neurobrucellosis can lead to significant morbidity [5], highlighting the importance of early recognition and appropriate treatment.

Neurobrucellosis manifests with a variety of clinical presentations. It has neither a typical clinical presentation nor a specific cerebrospinal fluid (CSF) profile or radiological findings for a clear clinical diagnosis [6]. This absence of distinctive features often leads to misdiagnosis and delay in diagnosis, complicating clinical management [7–9]. Diagnosis is usually made 2–12 months after symptom onset [8]. Despite its global prevalence, neurobrucellosis is often overlooked and underreported [7]. Therefore, a high level of suspicion is essential in endemic regions, and a comprehensive history of potential exposure should be taken. Patients presenting with neuropsychiatric symptoms or cognitive impairment should undergo thorough evaluation for neurobrucellosis and receive appropriate treatment [10].

Brucella can enter the CNS early in the disease through hematogenous spread, potentially causing latent or clinical meningitis and invading adjacent nervous structures. Neurobrucellosis can occur at any stage of systemic brucellosis, with early symptoms emerging during or shortly after the septicemic phase, while late symptoms, which are more common, can persist for months or even years. The exact mechanisms behind neurobrucellosis are not fully understood, but it may be due to the direct neurotoxic effects of the bacteria, the action of harmful cytokines or endotoxins, or the body’s immune and inflammatory responses. Cytotoxic lymphocytes and activated microglia play a role in the disease, and a weakened immune system can increase susceptibility. Additionally, persistent intracellular Brucella may trigger immune responses that contribute to neuropathology [4, 8].

Neurobrucellosis presents as acute meningitis or meningoencephalitis, chronic peripheral radiculopathy, or chronic meningoencephalitis. Diagnosis relies on isolating brucella from CSF or detecting anti-Brucella antibodies, though challenges include occasional negative serological results and variable culture sensitivity. Emerging techniques like CSF metagenomic sequencing are underutilized in endemic regions [1].

Given the limited data on long-term outcomes of neurobrucellosis, we performed a retrospective cohort study at two major referral centers in Mashhad, Iran, between March 21, 2011, and March 20, 2022. This study aimed to describe clinical characteristics, laboratory findings, neuroimaging features, and long-term morbidity and mortality associated with neurobrucellosis.

## Methods

This retrospective cohort study was conducted at two major referral centers for neuroinfections and neuroinflammations in Mashhad, Iran, the country’s second-largest city located in the northeast. The study included individuals who were diagnosed with neurobrucellosis between March 21, 2011, and March 20, 2022. Data were collected on age, gender, occupation, clinical features, radiological and laboratory findings, and clinical outcomes.

For patients admitted between March 2011 and September 2019, medical records and discharge letters were reviewed. For those admitted between October 2019 and March 2022, who were part of our registry of community-acquired suspected CNS infection, data were prospectively collected using an online patient registration form.

Neurobrucellosis in laboratory-confirmed brucellosis cases is diagnosed based on any of the following criteria:

1. suspected symptoms and signs of neurobrucellosis such as severe and persistent headache that disrupts normal activities, insomnia, confusion, depression, behavioral changes, incontinence, neck stiffness, and any neurological findings during examination;
2. isolation of Brucella from CSF and/or positive anti-Brucella antibodies in CSF;
3. presence of lymphocytic pleocytosis, elevated protein, and decreased glucose levels in CSF; or
4. abnormal findings on cranial MRI or CT scan [6].

All patients included in the study met the diagnostic criteria for brucellosis. Individuals with incomplete medical records or missing essential diagnostic information were excluded.

Neuroimaging (cranial MRI or CT) and CSF studies were not performed for all patients. The decision to perform these investigations was based on clinical judgment. In cases where the patient’s condition did not allow for these procedures, if they were contraindicated, or in the case of patient refusal, they were not conducted. Of the cohort, 90.6% (n = 96) underwent CSF analysis, while 76.4% (n = 81) underwent neuroimaging.

During the study period, conventional culture media were used for CSF culture, and standard BACTEC™ Plus Aerobic/F bottles were utilized for blood cultures. The conventional culture media were incubated for 14–21 days, while the BACTEC bottles were incubated for one week.

Following the Iranian national protocol for diagnosing and managing brucellosis in endemic areas, the cutoff titers were set at 1/80 for the Wright test, 1/40 for the Coombs test, and 1/40 for the 2-Mercaptoethanol (2ME) test, with other significant differential diagnoses being considered and ruled out. In CSF, a cutoff titer of 1/8 in the Wright test was deemed positive.

In cases where there was any doubt about the diagnosis, a thorough evaluation was conducted to exclude other potential causes of meningitis, especially when Brucella was not detected in the CSF, either microbiologically or serologically. This included testing for infectious etiologies like neurotuberculosis and cryptococcal meningitis, as well as considering non-infectious causes such as autoimmune and inflammatory disorders. The diagnostic approach followed the algorithm described in our prior study on subacute and chronic meningitis [11].

Patients diagnosed with neurobrucellosis were treated with a triple-drug regimen of ceftriaxone, doxycycline, and rifampin for 4 weeks, followed by doxycycline and rifampin for at least 4 months. For those who did not receive or complete a 4-week course of parenteral ceftriaxone, or did not tolerate one or more of these medications, or if they were contraindicated, trimethoprim–sulfamethoxazole (TMP/SMX) or other medications effective against brucella was included in the regimen.

For survivors discharged from the hospital, follow-up assessments were conducted via telephone at the conclusion of the study period to evaluate their survival and neuropsychological function based on self-reported outcomes. Clinical outcome was described as favorable and unfavorable based on Glasgow Outcome Scale (GOS) scores of 5 and 1–4, respectively [12].

### Statistics

Continuous data were summarized using medians and interquartile ranges, while categorical variables were represented by frequencies and percentages. The Mann–Whitney U test was employed for continuous variables, and Fisher’s exact test or Chi-square tests were utilized for categorical variables as appropriate. Significant predictors among the tested independent variables were identified through binary logistic regression analysis. Kaplan–Meier curves were applied for survival analysis. A *p*-value <0.05 was considered statistically significant throughout the analyses.

### Research ethics

All patients or their legally authorized representatives provided informed consent. This study received approval from the ethics committee of Mashhad University of Medical Sciences under the code IR.MUMS.REC.1400.263.

## Results

### Demographic and clinical characteristics

A total of 106 patients diagnosed with neurobrucellosis were evaluated. The median age of the patients was 30 years (IQR: 21.8–46.3) (age range: 4–87 years), with 8 patients (7.5%) being elderly (≥65 years) and 20 patients (18.9%) being pediatric (under 18 years). The majority of the patients were male (63/106, 59.4%) (Table 1).

**Table 1:** Characteristics of patients with neurobrucellosis

Variables	Total (n = 106)
Age (years)	30 (21.8–46.3)^#^
Elderly (≥65 years)	8/106 (7.5)
Pediatric (<18 years)	20/106 (18.9)
Gender (male)	63/106 (59.4)
Underlying comorbidities	
Cardiovascular disorders	12/106 (11.3)
Immunocompromised^*^	6/106 (5.7)
Diabetes mellitus	3/106 (2.8)
Rheumatologic disorders	1/106 (0.9)
Multiple sclerosis	1/106 (0.9)
HIV/AIDS	1/106 (0.9)
CKD/ESRD	1/106 (0.9)
Cancer	0
Organ transplant	0
Addiction	12/106 (11.3)
Clinical manifestations	
Headache	82/106 (77.4)
Fever	79/106 (74.5)
Musculoskeletal pain	58/101 (57.4)
FNDs	
Cranial nerve palsies	30/105 (28.6)
CN I	1
CNII	3
CN III	3
CN IV	2
CN V	3
CN VI	8
CN VII	4
CN VIII	9
CN XII	1
Multiple cranial nerve palsies	10
FNDs other than cranial nerve palsies	42/105 (40)
Meningeal signs	47/106 (44.3)
Sweating	41/91 (45.1)
Visual problem	35/106 (33)
Paresis	28/106 (26.4)
Altered level of consciousness	20/106 (18.9)
Hallucination/delusion/disorganized speech	18/106 (17)
Seizures	16/106 (15.1)
Papilledema	10/100 (10)
Speech problem	10/103 (9.7)
Classic triad of meningitis^1^	11/106 (10.4)
Classic triad of headache, fever, and meningeal signs	37/106 (34.9)
Peripheral neuropathy, radiculopathy, or Guillain Barre syndrome	7/106 ()
Extra-nervous system involvement	19/101 (18.8)
Symptom duration (days)	14 (7–30)
Neuroimaging findings	
Normal	50/82 (60.9)
White matter changes in brain or spinal cord	17/82 (20.7)
Hydrocephalus	2/82 (2.4)
Meningeal enhancement	11/82 (13.4)
Cerebral infarction	8/82 (9.8)
Space occupying lesion	4/82 (4.9)
Laboratory features	
CSF leukocytes, per µl	80 (10–200)
CSF Lymphocyte predominance^2^	46/83 (55.4)
CSF protein, mg/dl	81.4 (49–157)
CSF glucose, mg/dl	41 (23–59)
Serum Wright (STA)	1/320 (1/160–1/640)
Serum 2ME	1/160 (1/80–1/320)
Serum Coombs-Wright	1/320 (1/80–1/320)
CSF Wright	1/64 (1/20–1/160)
Positive CSF serology for brucella	23/48 (47.9)
Positive CSF or blood culture for brucella	6/80 (7.5)
Length of hospital stay (days)	11 (7–19.8)
Duration of in-hospital treatment (days)	9 (6–16.5)
In-hospital mortality	5/106 (4.7)
Duration of follow up (months)	52 (35–77)
Unfavorable outcome (at the end of follow-up) (GOS)	20/85 (23.5)

*HIV* human immunodeficiency virus, *AIDS* acquired immunodeficiency syndrome, *CKD/ESRD* end-stage renal disease/chronic kidney disease, *FNDs* focal neurologic deficits, *CSF* cerebrospinal fluid

^#^Numbers are represented by the number of patients with specific characteristics/ the number of patients evaluated (percent) for nominal variables and by median (25th quartile, 75th quartile) for quantitative variables

^*^Diabetes mellitus (3), HIV (1), nephrotic syndrome taking immunosuppressive medications (1), antiphospholipid syndrome (1)

^1^Characterized by fever, altered consciousness, and neck stiffness

^2^Defined as comprising more than 50 percent lymphocytes in cerebrospinal fluid

### Underlying comorbidities

The prevalence of underlying comorbidities was as follows: 12 (11.3%) had cardiovascular disorders, 6 patients (5.7%) were immunocompromised, 3 (2.8%) had diabetes mellitus, 1 (0.9%) had rheumatologic disorders, 1 (0.9%) had HIV/AIDS, and 1 (0.9%) had chronic kidney disease/end-stage renal disease (CKD/ESRD). No patients had cancer or organ transplants. Drug addiction was noted in 12 patients (11.3%).

### Clinical manifestations

Common clinical manifestations included headache in 82 patients (77.4%), fever in 79 patients (74.5%), musculoskeletal pain in 58 of 101 patients (57.4%), and night sweats in 41 of 91 patients (45.1%). Seizures were reported in 16 patients (15.1%), and altered level of consciousness in 20 patients (18.9%). Meningeal signs were identified in 47 (44.3%) of 106 patients. The median symptom duration before the onset of neurologic symptoms was 14 days (interquartile range [IQR], 7–30 days).

Focal neurological deficits (FNDs) were present in 57 of 105 patients (54.3%), with cranial nerve palsies occurring in 30 patients (28.6%). FNDs other than cranial nerve palsies were observed in 42 patients (40%). Visual problems were reported by 35 patients (33%), and speech problems by 10 of 103 patients (9.7%). Paresis was noted in 28 patients (26.4%), while hallucinations, delusions, or disorganized speech were observed in 18 patients (17%).

In our study, 19 (18.8%) of 101 patients had concomitant extra-nervous system involvement. The most common were ten cases of osteoarticular involvement, including five cases of vertebral osteomyelitis. This was followed by three cases of epididymoorchitis, one case of an aortic aneurysm, one case of skin vasculitis, one case of myositis, one case of conjunctivitis, and one case of Brucella endocarditis.

### Neuroimaging findings

Neuroimaging findings showed white matter changes in the brain in 17 of 82 patients (20.7%). Meningeal enhancement was seen in 11 patients (13.4%), cerebral infarction in 8 patients (9.8%), and abscess/granuloma in 4 patients (4.9%). Hydrocephalus was identified in 2 (2.4%) patients.

There was one patient with subdural hematoma and one with cervical spine epidural abscess. Brain angiography in another patient with advanced HIV infection and concomitant ophthalmic zoster showed numerous cerebral aneurysm and brain infarction.

Overall, 50 of 82 (60.9%) patients had normal neuroimaging.

### Laboratory features

CSF analysis revealed pleocytosis with a median CSF leukocyte count of 80 per µl (IQR: 10–200). CSF lymphocyte predominance was observed in 46 of 83 patients (55.4%). The median CSF protein level was 81.4 mg/dl (IQR: 49–157), and CSF hypoglycorrhachia was present in 42 out of 91 patients (46.2%), with a median CSF glucose level of 41 mg/dl (IQR: 23–59).

Serum Wright (STA) test had a median titer of 1/320 (IQR: 1/160–1/640), Serum 2ME had a median titer of 1/160 (IQR: 1/80–1/320), and Serum Coombs-Wright had a median titer of 1/320 (IQR: 1/80–1/320). CSF Wright test had a median titer of 1/64 (IQR: 1/20–1/160). Positive CSF serology for Brucella was found in 23 of 48 patients (47.9%), while positive CSF or blood culture for Brucella was seen in 6 of 80 patients (7.5%).

### Hospitalization and outcomes

The median length of hospital stay was 11 days (IQR: 7–19.8), and the median duration of in-hospital treatment was 9 days (IQR: 6–16.5). In-hospital mortality was reported in 5 patients (4.7%). The median duration of follow-up was 52 months (IQR: 35–77). Overall, 20 (23.5%) of 85 patients experienced an unfavorable outcome based on the Glasgow Outcome Scale (GOS).

### Treatment

Table 2 summarizes the antibiotic treatments administered to patients with neurobrucellosis. Ceftriaxone was used in 84 of 101 patients (83.2%), doxycycline in 86 of 102 patients (84.3%), and rifampin in 87 of 101 patients (86.1%). TMP/SMX was administered to 36 of 101 patients (35.6%), gentamicin to 20 of 100 patients (20%), and streptomycin to 9 of 102 patients (8.8%). Fluoroquinolones were used in 8 of 99 patients (8.1%).

**Table 2:** Antibiotic treatments administered to patients with neurobrucellosis

Antibiotics	
Rifampin	87/101 (86.1)^#^
Doxycycline	86/102 (84.3)
Ceftriaxone	84/101 (83.2)
TMP/SMX	36/101 (35.6)
Gentamicin	20/100 (20)
Streptomycin	9/102 (8.8)
Fluoroquinolone	8/99 (8.1)
Any dose of corticosteroids	48/100 (48)
Major complications of the treatment	
Nephrotoxicity	8/101 (7.9)
Drug eruption^*^	6/101 ()
Hepatitis	4/101 (4)
Esophagitis	1/101 ()

*TMP/SMX* Trimethoprim/Sulfamethoxazole

^*^Including one case of Stevens-Johnson syndrome

^#^ Numbers are represented by the number of patients with specific characteristics/ the number of patients evaluated (percent) for nominal variables

Corticosteroids were administered to 48 out of 100 patients (48%), including even a single dose, at some point during their treatment.

### Major complications of the treatment

Several major complications were observed in the treatment of neurobrucellosis. Nephrotoxicity occurred in 8 of 101 patients (7.9%), and hepatitis in 4 patients (4%). There was 1 case of esophagitis, and 6 patients experienced drug eruptions, including one case of Stevens-Johnson syndrome.

### Long-term complications

The most common long-term complications included blindness, hearing problems, ataxia or vertigo, and chronic headache, each affecting three (3.5%) patients. Hemifacial paresis, memory impairment, and limb paresthesia were observed in two (2.4%) patients each. Other complications included sexual dysfunction, hand tremor, urinary incontinence, and seizure, each affecting one (1.2%) patient.

### Predictors of mortality

The univariable analysis showed that older age was significantly associated with increased mortality (*p* = 0.008, OR = 1.048, 95% CI: 1.012–1.085). Altered level of consciousness (*p* = 0.020, OR = 5.400, 95% CI: 1.391–20.966) and seizures (*p* = 0.007, OR = 7.727, 95% CI: 1.925–31.012) were also significantly linked to higher mortality. The absence of meningeal signs was associated with a lower risk of mortality (OR = 0.121, 95% CI: 0.015–0.991, *p* = 0.040). Elevated body temperature on admission was a significant predictor of mortality (*p* = 0.041, OR = 2.142, 95% CI: 1.031–4.452), as were white matter changes on neuroimaging (*p* = 0.028, OR = 5.000, 95% CI: 1.250–19.992). Additionally, higher CSF glucose levels were linked to increased mortality (*p* = 0.036, OR = 1.029, 95% CI: 1.002–1.057) (Table 3).

**Table 3:** Predictors of mortality

	Yes	No	Univariate analysis^*^		
			*P*-value	Odds ratio	95% CI
Age			0.008	1.048	1.012–1.085
Altered level of consciousness	5/20 (25)^#^	5/86 (5.8)	0.020	5.400	1.391–20.966
Seizure	5/16 (31.2)	5/90 (5.6)	0.007	7.727	1.925–31.012
Meningeal signs	1/47 (2.1)	9/59 (15.3)	0.040	0.121	0.015–0.991
Body temperature on admission			0.041	2.142	1.031–4.452
White matter changes in brain	5/17 (29.4)	5/65 (7.7)	0.028	5.000	1.250–19.992
CSF glucose			0.036	1.029	1.002–1.057
Corticosteroid	8/48 (16.7)	2/52 (3.8)	0.045	5.000	1.005–24.872
LOS			0.004	1.065	1.021–1.112

*LOS* Length of hospital Stay, *CSF* Cerebrospinal Fluid

^#^Numbers are represented by the number of patients with specific characteristics/ the number of patients evaluated (percent) for nominal variables and by median (25th quartile, 75th quartile) for quantitative variables

^*^The analysis was conducted using binary logistic regression

### Survival analysis

**Fig. 1:**
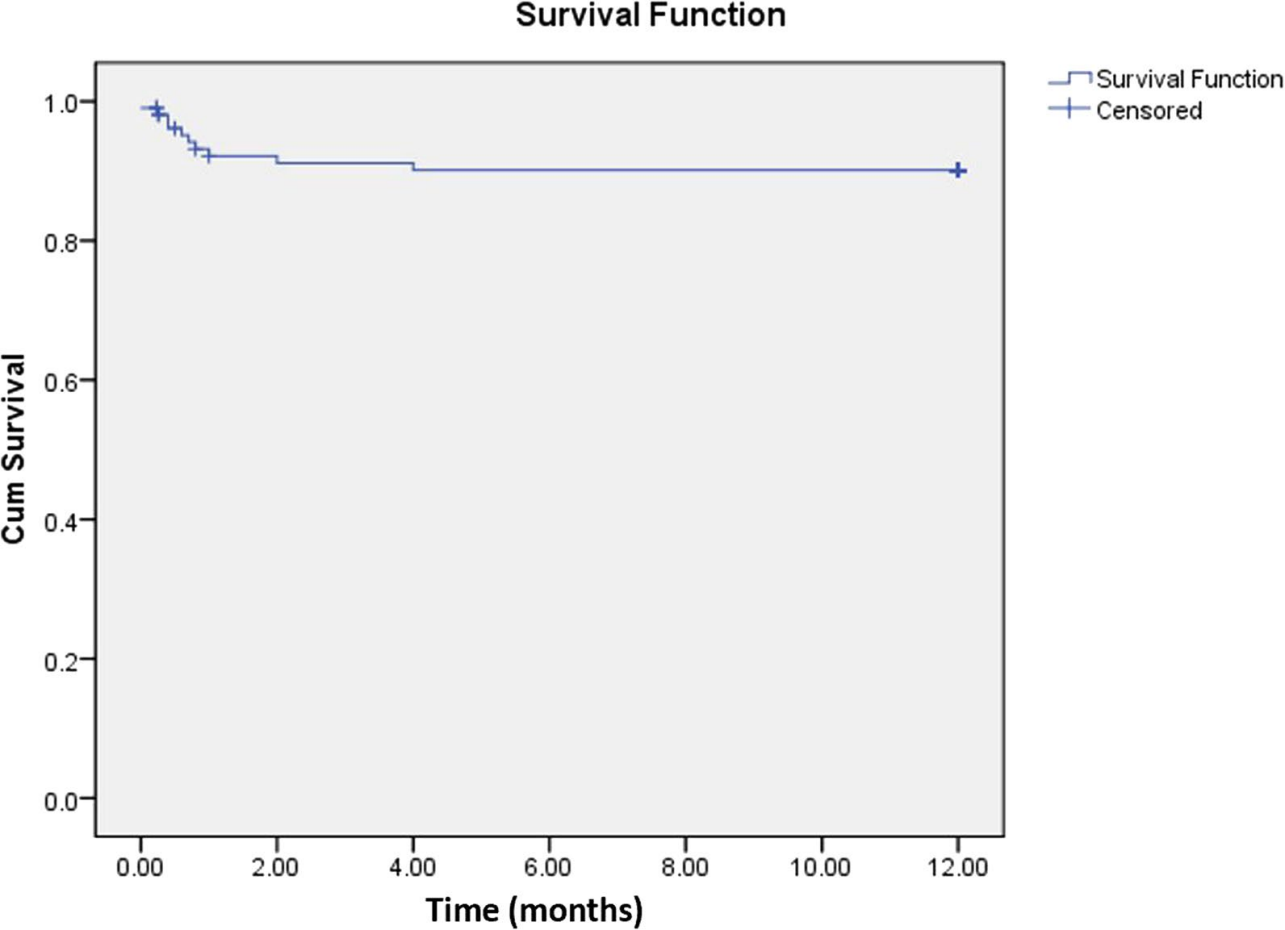
Kaplan–Meier survival curve for patients with neurobrucellosis. This curve illustrates the survival probability over time for patients diagnosed with neurobrucellosis. The x-axis represents time (months) from hospital admission to death or last follow-up, while the y-axis shows the survival probability

The survival outcomes of the cohort were evaluated using Kaplan–Meier analysis, with follow-up units representing monthly intervals (Fig. 1). Events recorded within the study period denote patient deaths.

At 1 months since hospital admission, the survival estimate was 0.922 (SE = 0.027), indicating that approximately 92.2% of patients survived up to this point. A total of 8 events were recorded by the end of this period.

At 6 months since hospital admission, the survival estimates further decreased to 0.901 (SE = 0.030), indicating that 90.1% of patients survived up to one year. A total of 10 events were recorded by the end of the observation period.

No additional deaths were recorded after the initial 4 months of follow-up, suggesting a period of stability in patient outcomes after the first 6 months.

## Discussion

Our study provides a comprehensive analysis of the clinical characteristics, treatment outcomes, and survival rates of patients diagnosed with neurobrucellosis. Notably, it is one of the largest cohorts of neurobrucellosis patients studied worldwide, offering valuable insights into this challenging condition. The cumulative survival rate in our cohort was 92.2% at one month and 90.1% at six months since hospital admission, indicating that most fatal outcomes occurred within the first four months, after which survival rates stabilized. This highlights the need for vigilant monitoring and intervention during this critical period to improve patient outcomes. Mortality rates for neurobrucellosis reported in the literature range from 0 to 27% [6]. Long-term neurological sequelae were observed in one out of every six survivors in our study, underscoring the potential for persistent neurological damage despite treatment. Overall, 23% of patients in our study experienced unfavorable outcomes, based on GOS. Long-term complications observed during follow-up commonly included blindness, hearing issues, hemifacial paresis, and memory impairment. These findings underscore the substantial impact of neurobrucellosis on survivors’ quality of life and highlight the debilitating nature of these complications, emphasizing the ongoing need for clinical support and rehabilitation for affected individuals. It aligns with previous studies that reported long-term sequelae despite appropriate antibiotic therapy. For example, in a study of 215 patients with neurobrucellosis (The Istanbul study), one fifth of treated patients experienced permanent sequelae, with walking difficulty, hearing loss, and urinary incontinence being the most frequent permanent functional losses seen after treatment of the disease [5].

The univariable analysis of mortality predictors in neurobrucellosis patients identifies several significant factors that underscore the disease’s severity. Older age was significantly associated with higher mortality (*p* = 0.008, OR = 1.048), aligning with the general trend of poorer outcomes in older individuals due to weakened immune responses and comorbidities. Seizures (*p* = 0.007, OR = 7.727) and altered levels of consciousness (*p* = 0.020, OR = 5.400) were also strongly linked to increased mortality. In addition, the absence of meningeal signs was linked to a lower risk of mortality (OR = 0.121, 95% CI: 0.015–0.991, *p* = 0.040). White matter changes observed on neuroimaging (*p* = 0.028, OR = 5.000) were similarly associated with a poor prognosis. In addition to neurological symptoms and imaging findings, elevated body temperature on admission (*p* = 0.041, OR = 2.142) emerged as a significant predictor of mortality. Interestingly, higher cerebrospinal fluid (CSF) glucose levels were associated with increased mortality (*p* = 0.036, OR = 1.029), which contrasts with typical trends observed in bacterial infections [13]. Further investigation is needed to validate this observation.

Neurobrucellosis can present with a variety of clinical manifestations, which in our study primarily included headache (77.4%), fever (74.5%), and musculoskeletal pain (57.4%). These findings are consistent with the literature, which highlights these symptoms as prevalent in neurobrucellosis patients [14]. Although the classic triad of meningitis (fever, altered consciousness, and meningeal signs) was only observed in 10% of patients, the triad of fever, headache, and meningeal signs was observed in one third of patients. In our study, about one-third of the patients complained of visual problems, and about 15% developed seizures. One young boy presented with New Onset Refractory Status Epilepticus (NORSE) and required intubation. Initially, due to hallucinations of an animal bite, rabies was suspected. However, he was ultimately diagnosed with neurobrucellosis and made a full recovery. Although neurobrucellosis has been rarely reported in children [15], about one-fifth of patients in our cohort were in the pediatric age range. This highlights the importance of considering neurobrucellosis in pediatric differential diagnoses, particularly in endemic areas.

We also identified a rare relapsing–remitting presentation of neurobrucellosis in two cases. In one, symptoms initially presumed to be viral meningitis appeared severely over a few days, then subsided, continuing with psychiatric symptoms. Three years later, similar severe symptoms recurred. The first time, the CSF Wright test turned positive after the patient’s discharge, and during the second episode, it became positive again, confirming the diagnosis of neurobrucellosis. In the second case, the patient developed a meningeal syndrome, initially attributed to a large arachnoid cyst found on brain CT scan, which prompted craniotomy. However, 3-month later, similar symptoms reappeared, confirmed this time to be caused by neurobrucellosis. The mechanism behind the relapsing–remitting presentation of neurobrucellosis in these cases remains unclear but could involve several hypotheses. One possibility is bacterial persistence, where Brucella species evade initial immune responses and persist within host cells, potentially reactivating symptoms intermittently. Another consideration is the variability in host immune responses, which may lead to cycles of inflammation and quiescence in response to the infection. These cases underscore the intricate nature of neurobrucellosis, where the interplay between bacterial persistence and host immune factors likely influences the disease’s relapsing pattern. They also highlight the complexity and variability of neurobrucellosis presentations, highlighting the importance of considering it in differential diagnoses, especially in endemic areas or in patients with recurrent or atypical CNS symptoms.

Cranial nerve involvement is also common; in our study, approximately 29% of patients had affected cranial nerves. However, reports have indicated that the frequency can be over 70% [3]. Typically, one or more cranial nerves are involved, primarily as a result of basal meningitis. However, cranial nerve involvement can also result from potential vasculitic processes, pseudotumor cerebri, or the side effects of tetracycline, which is frequently used to treat the disease. Cranial nerve paralysis is more commonly observed during the acute or subacute phases of the illness when the CNS is involved. However, isolated cranial nerve involvement has also been reported, though it is rare [4]. The vestibulocochlear nerve is the most frequently affected cranial nerve in neurobrucellosis, resulting in sensorineural hearing loss [14]. Other cranial nerves frequently involved in neurobrucellosis include the trigeminal, facial, abducens, oculomotor, and optic nerves, either individually or in combination. In our study, multiple cranial nerve palsies were observed in 10% of cases, with the eighth and sixth cranial nerves being the most commonly affected, followed by the seventh cranial nerve.

The exact mechanism of vestibulocochlear involvement in neurobrucellosis is unclear, but it is believed to involve the direct invasion and penetration of Brucella endotoxin, which may cause damage and impairment to the labyrinth and vestibulocochlear nerve function [16]. Hearing loss resulting from vestibulocochlear nerve involvement has been frequently reported as a distinct feature of neurobrucellosis, distinguishing it from neurotuberculosis. Consequently, electrophysiological studies have been recommended to identify subclinical vestibulocochlear nerve involvement, as they can aid in diagnosing neurobrucellosis [1]. The abducens nerve, also known as CN VI, travels the longest path within the skull and is particularly vulnerable to injury. Abducens nerve palsy may be associated with inflammation of the intracranial meninges. Brucella’s ability to impact Schwann cells upon entering the neuraxis via the circulatory or lymphatic systems can result in nerve demyelination. Additionally, vascular inflammation likely contributes to the development of abducens nerve palsy. Notably, abducens nerve palsy can precede the onset of meningitis symptoms in patients with brucellosis, suggesting that cranial nerve involvement may occur prior to meningeal infection [17]. Isolated cranial nerve involvement is a rare condition, with the vestibulocochlear nerve being the most commonly affected. There have been a few instances of isolated abducens palsy reported [18].

Additionally, neurobrucellosis can cause complications in the arachnoid drainage system, leading to intracranial hypertension [8]. Pseudotumor cerebri is an uncommon manifestation of neurobrucellosis. It is noteworthy that tetracyclines and their derivatives can mimic the symptoms of pseudotumor cerebri. Therefore, before attributing pseudotumor cerebri to brucellosis, it is crucial to confirm that the patient is not using these medications or their derivatives [19]. One patient in our study developed hydrocephalus severe enough to require CSF shunt placement, and another patient showed severe intracranial hypertension that did not resolve with anti-brucella medication and required long-term corticosteroid treatment for resolution. The second case also exhibited worsening of symptoms under treatment with anti-brucella medication, which resembled a paradoxical reaction. The mechanism of pseudotumor cerebri in neurobrucellosis is not fully elucidated in the available literature. Pseudotumor cerebri, or idiopathic intracranial hypertension, is characterized by increased intracranial pressure without an identifiable structural cause. In the context of neurobrucellosis, it is hypothesized that this condition may arise due to inflammation and involvement of the meninges or arachnoid granulations by Brucella organisms that could obstruct the normal flow of CSF, leading to increased pressure within the cranial cavity.

Peripheral nervous system (PNS) involvement has been reported in approximately 7% of neurobrucellosis cases, a finding consistent with our study. Case reports have documented a range of polyradiculoneuropathies, including acute, subacute, and chronic forms, some presenting without sensory symptoms or resembling Guillain-Barré syndrome [8, 20, 21]. Demyelination in peripheral nerves has been noted, with polyradiculitis often presenting as a progressively worsening flaccid paralysis that mainly affects the legs, while upper limb involvement is rare. Literature suggests that patients with peripheral neuropathy tend to be older and experience a longer duration of symptoms compared to those without neuropathy, though these differences are not statistically significant. Brucella organisms are known for their prolonged intracellular survival within phagocytes, which might impact the PNS. However, the mechanisms by which these bacteria cause peripheral nerve dysfunction and its reversibility remain unclear. The direct effects of Brucella or its endotoxins on peripheral nerves, combined with immune-mediated damage, may contribute to peripheral neuropathy [22].

Brucella infection can impact the spinal cord by potentially causing abscesses and granulomas that compress it, or by affecting the spinal roots [23]. Additionally, cases of myelopathy have been documented, including reports of transverse myelitis and spinal arachnoiditis [15]. In cases of brucellosis, spinal abscesses or granulomas can lead to upper motor neuron-type lesions, while involvement of spinal roots by Brucella may result in lower motor neuron-type lesions [23]. Our study included a 57-year-old patient who developed a brucellar cervical spine epidural abscess, resulting in cord compression.

The most common long-term sequelae among our patients were blindness and hearing impairments. Brucella can damage the optic nerve, often presenting as optic disc edema [17]. Both pseudotumor cerebri and papillitis (optic neuritis) are involved in the pathophysiology of optic disc edema. Pseudotumor cerebri is characterized by increased CSF pressure and papilledema, while generally preserving vision and pupillary reflexes. In contrast, papillitis (optic neuritis) presents with eye movement pain, optic disc edema, rapid visual loss, and a relative afferent pupillary defect [4]. Optic disc edema in brucellosis can result from axoplasmic congestion of the optic nerve’s unmyelinated axons, secondary to inflammation and demyelination behind the optic nerve head. Optic nerve edema due to vasculitis, a hallmark of brucellosis, typically has a poor prognosis, often progressing to optic nerve atrophy despite treatment efforts. Conversely, papilledema caused by increased intracranial pressure from Brucella infection usually has less impact on visual acuity and pupillary reflexes compared to vasculitis-induced optic neuritis, which may lead to significant vision loss and afferent pupillary disturbances [17]. In our study, 10% of patients had optic disc swelling. Based on the information provided, it could be suggested that in patients with neurobrucellosis presenting with optic disc edema, measuring intracranial pressure (ICP) should be considered. If the ICP is found to be within the normal range, particularly in those with significant visual impairments, it may be prudent to prescribe corticosteroids in addition to standard anti-brucella medications. This approach could help manage potential inflammatory or demyelinating effects contributing to optic nerve damage and mitigate long-term sequelae such as blindness.

Neurobrucellosis presents four types of imaging abnormalities: normal, inflammation, white matter changes, and vascular insults. Inflammation can cause granulomas or enhance the meninges, perivascular spaces, or lumbar nerve roots. Inflammatory signs typically resolve on follow-up MR or CT images after appropriate treatment, leading to full clinical recovery. Granulomatous formations, especially in the sellar region, are rare but show clinical and radiologic improvement after a few months of treatment. White matter changes in neurobrucellosis appear as hyperintense lesions on T2-weighted images and manifest in three patterns: diffuse, periventricular, and focal demyelinating. The exact cause of these changes is unclear, but they might be due to autoimmune reactions. These white matter changes can mimic other conditions like multiple sclerosis, acute disseminated encephalomyelitis (ADEM), or Lyme disease. Involvement of the corpus callosum and intraparenchymal subcortical enhancing lesions are rare in neurobrucellosis [24]. In our study, white matter lesions were relatively common, identified in about 20% of patients. These lesions were associated with a higher risk of death among patients with neurobrucellosis. White matter lesions may persist despite significant clinical improvement and normalization of CSF following adequate treatment [24]. It has been noted that white matter changes in neurobrucellosis result from demyelination, as confirmed by pathological studies [25]. Autopsy evidence further supports this, showing demyelination resembling lesions seen in multiple sclerosis in neurobrucellosis patients. These findings suggest that white matter involvement in neurobrucellosis may arise from an immune-mediated response to Brucella infection within the CNS [24].

Neurobrucellosis can lead to a range of meningovascular complications, such as mycotic aneurysms, ischemic strokes, and subarachnoid hemorrhages. Vascular damage in neurobrucellosis is likely attributable to two mechanisms. Firstly, an inflammatory process affecting small vessels or the venous system can lead to lacunar infarcts, small hemorrhages, or venous thromboses. Secondly, hemorrhagic stroke may result from the rupture of a mycotic aneurysm, a potential consequence of embolic stroke related to brucellar endocarditis [24]. Transient ischemic attacks (TIA) and ischemic strokes in neurobrucellosis are not fully understood but may result from vascular inflammation, spasm, or embolism. Although it has been mentioned that this disease does not show a predilection for the size or location of vascular structures and can affect both arterial and venous systems [15], large vessel involvement is rare. Ischemic events are likely due to vasculitis. Carotid angiograms may reveal diffuse vascular spasm, yet many patients with ischemic symptoms present with normal angiograms. Diffusion-weighted imaging is useful for the early detection of infarctions, often revealing multiple lacunar infarcts in areas supplied by deep penetrating vessels, such as the brainstem, basal ganglia, and white matter [4]. Neuroimaging revealed that about 10% of patients in our study had cerebral infarctions. There was an HIV-infected patient in our study who presented with multiple mycotic aneurysms, concurrently suffering from ophthalmic zoster, and Brucella was isolated from the CSF by culture. Both infections, brucellosis and herpes zoster, can independently cause such vascular lesions. The co-occurrence of these conditions raises the possibility of an additive effect, further complicating the clinical presentation and management. This case also underscores the importance of considering multiple concurrent infections in immunocompromised patients.

In diagnosing brucellosis, the gold standard is bacterial isolation from serum and other specimens. However, many reports indicate that culture positivity is less than 50% [15]. In comparison, our study showed that, although less than half of the patients were tested for CSF Wright, 47.9% of those tested had positive results. Additionally, only 7.5% had positive CSF or blood cultures for Brucella. These findings highlight a significant diagnostic challenge in neurobrucellosis, where traditional methods such as culture may not always be reliable. The low culture positivity rate underscores the need for alternative diagnostic approaches or adjunctive tests to improve detection rates. The high percentage of positive CSF Wright tests suggests that serological methods can play a crucial role in the diagnosis of neurobrucellosis, particularly when cultures fail to yield results. Published studies have demonstrated that Brucella PCR in CSF has a sensitivity of 100%, surpassing culture in all cases. However, despite its promise, there is still a lack of sufficiently powered studies to confirm its effectiveness [26]. Additionally, research by Guven et al. (2013) found that lumbar puncture in 128 laboratory-confirmed brucellosis cases with neurological symptoms revealed that 48 (37.5%) were diagnosed with neurobrucellosis, demonstrating CSF Wright’s high sensitivity (0.94), specificity (0.96), and positive and negative predictive values of 0.94 and 0.96, respectively. These results indicate that serological methods, particularly CSF Wright, can be highly effective in diagnosing neurobrucellosis and may offer a valuable complement to culture-based techniques [6]. Further research is needed to evaluate the efficacy of combining serological and molecular diagnostic techniques to enhance the detection of Brucella in clinical practice and improve overall diagnostic accuracy for this challenging condition.

Regarding the treatment of brucellosis, monotherapy can lead to relapses; thus, combination therapy is recommended [15]. Various studies have identified ceftriaxone as the most effective extended-spectrum cephalosporin for treating Brucella species. However, using cephalosporins alone in brucellosis patients has often led to frequent treatment failures and relapses. This is likely because ceftriaxone can diffuse into phagocytes but does not accumulate within them. According to a study by Erdem et al. [5], ceftriaxone-based regimens achieved significantly higher therapeutic success compared to oral regimens when considering treatment failures or relapses as negative outcomes. For neurobrucellosis, the recommended treatment duration is several months based on the patient’s response. In practice, the average treatment duration has been approximately 4–6 months or longer. Doxycycline is the tetracycline of choice for neurobrucellosis because it penetrates tissues and the CNS more effectively and has a longer half-life [15]. The antibiotics most commonly used in our study were ceftriaxone (83.2%), doxycycline (84.3%), and rifampin (86.1%), reflecting standard treatment protocols for neurobrucellosis due to their efficacy in penetrating the CNS. TMP/SMX was also used in about one-third of patients. However, complications such as nephrotoxicity (7.9%) highlight the need for careful monitoring during treatment.

Although this study represents one of the largest cohorts of patients with neurobrucellosis, it has several limitations. First, because a significant portion of the patients was identified retrospectively, detailed clinical information was not available for all cases. Second, the relatively small sample size limited the study’s statistical power, resulting in a wide range of interval estimations when significant results were found, thus making the findings less conclusive.

## Conclusion

In conclusion, neurobrucellosis presents significant diagnostic and therapeutic challenges, with a wide range of clinical manifestations and potential for long-term complications. Given the diverse clinical and paraclinical presentations of neurobrucellosis, early and accurate diagnosis is vital for effective treatment and better outcomes. While no single sign or symptom is definitive, a combination of clinical findings and supporting paraclinical data should raise suspicion, particularly in patients with relevant clinical backgrounds and epidemiological risk factors. Clinicians should maintain a high index of suspicion and know when to test for neurobrucellosis, especially in individuals with unexplained neuropsychiatric symptoms, those in endemic regions, or those with a history of brucellosis or potential exposure to Brucella. Neurobrucellosis should be considered in cases of meningitis with meningo-vascular complications, cranial neuropathies, visual disturbances, or white matter changes on neuroimaging. While less common, pediatric cases, which account for about 20% of diagnoses, should not be overlooked. Additionally, testing is warranted in patients with systemic brucellosis who develop neuropsychiatric complications. The first-year post-diagnosis is critical, and early identification of significant predictors of mortality such as older age, altered consciousness, seizures, and white matter changes can guide therapeutic interventions to improve patient outcomes. Future studies with larger cohorts and standardized follow-up protocols are needed to further elucidate the natural history and optimal management strategies for neurobrucellosis.

## Supplementary Information

Additional file 1.

## Data Availability

No datasets were generated or analysed during the current study.
