# Supplementary material for: Neurobrucellosis: a retrospective cohort of 106 patients
Source: Trop Med Health. 2025 Jan 15;53:9. doi: 10.1186/s41182-025-00680-1 (PMC11737133; doi:10.1186/s41182-025-00680-1)
Supplement: Supplementary file 1 — Additional file 1. [file 41182_2025_680_MOESM1_ESM.docx]

**Supplementary table 1. Predictors of mortality (full table)**

|  |  |  | Univariable analysis^*^ | | |
| --- | --- | --- | --- | --- | --- |
|  |  |  | P-value | Odds Ratio | 95% CI |
|  | Yes | No |  |  |  |
| Age |  |  | **0.008** | 1.048 | 1.012-1.085 |
| Sex, male | 7/63 (11.1) ^#^ | 3/43 (7) | 0.737 | 1.667 | 0.406-6.841 |
| Addiction | 3/12 (25) | 7/94 (7.4) | 0.085 | 4.143 | 0.909-18.879 |
| Duration of symptoms |  |  | 0.684 | 0.994 | 0.964-1.025 |
| Headache | 7/82 (8.5) | 3/24 (12.5) | 0.691 | 0.653 | 0.155-2.748 |
| Fever | 7/79 (8.9) | 3/27 (11.1) | 0.713 | 0.778 | 0.186-3.248 |
| Altered level of consciousness | 5/20 (25) | 5/86 (5.8) | **0.020** | 5.400 | 1.391-20.966 |
| Seizure | 5/16 (31.2) | 5/90 (5.6) | **0.007** | 7.727 | 1.925-31.012 |
| Visual problem | 1/35 (2.9) | 9/71 (12.7) | 0.160 | 0.203 | 0.025-1.668 |
| Speech problem | 2/10 (20) | 8/93 (8.6) | 0.250 | 2.656 | 0.480-14.696 |
| FND other than cranial nerve palsies | 6/42 (14.3) | 3/63 (4.8) | 0.151 | 3.333 | 0.785-14.156 |
| Cranial nerve palsies | 1/30 (3.3) | 8/75 (10.7) | 0.441 | 0.289 | 0.035-2.416 |
| Meningeal signs | 1/47 (2.1) | 9/59 (15.3) | **0.040** | 0.121 | 0.015-0.991 |
| Body temperature on admission |  |  | **0.041** | 2.142 | 1.031-4.452 |
| White matter changes in brain | 5/17 (29.4) | 5/65 (7.7) | **0.028** | 5.000 | 1.250-19.992 |
| Meningeal enhancement | 3/11 (27.3) | 7/71 (9.9) | 0.128 | 3.429 | 0.736-15.982 |
| Brain infarct | 2/8 (25) | 8/74 (10.8) | 0.251 | 2.750 | 0.473-15.992 |
| Extra-nervous system involvement | 2/19 (10.5) | 7/82 (8.5) | 0.676 | 1.261 | 0.240-6.612 |
| CSF leukocytes |  |  | 0.241 | 0.992 | 0.979-1.005 |
| CSF polymorphonuclears |  |  | 0.317 | 1.018 | 0.983-1.053 |
| CSF protein |  |  | 0.644 | 0.999 | 0.992-1.005 |
| CSF glucose |  |  | **0.036** | 1.029 | 1.002-1.057 |
| Serum Wright |  |  | 0.401 | 1.001 | 0.999-1.002 |
| Serum 2ME |  |  | 0.644 | 0.999 | 0.995-1.003 |
| Ceftriaxone | 9/84 (10.7) | 1/17 (5.9) | 1.000 | 1.920 | 0.227-16.243 |
| Doxycycline | 9/86 (10.5) | 1/16 (6.2) | 1.000 | 1.753 | 0.207-14.883 |
| Rifampin | 7/87 (8) | 3/14 (21.4) | 0.141 | 0.321 | 0.072-1.427 |
| TMP/SMX | 5/36 (13.9) | 5/65 (7.7) | 0.323 | 1.935 | 0.521-7.196 |
| Gentamicin | 4/20 (20) | 6/80 (7.5) | 0.110 | 3.083 | 0.779-12.204 |
| Corticosteroid | 8/48 (16.7) | 2/52 (3.8) | **0.045** | 5.000 | 1.005-24.872 |
| Treatment duration in hospital |  |  | 0.209 | 1.034 | 0.982-1.089 |
| LOS |  |  | **0.004** | 1.065 | 1.021-1.112 |

LOS: Length of hospital Stay; CSF: Cerebrospinal Fluid; FND: Focal Neurological Deficit; 2ME: 2-Mercaptoethanol; TMP/SMX: Trimethoprim/Sulfamethoxazole.

#Numbers are represented by the number of patients with specific characteristics/ the number of patients evaluated (percent) for nominal variables and by median (25^th^ quartile, 75^th^ quartile) for quantitative variables.

*The analysis was conducted using binary logistic regression.
